# Clinical performance of light-cured orthodontic adhesives for bonding brackets – an *in-vitro* study.

**DOI:** 10.12688/f1000research.141328.1

**Published:** 2023-11-07

**Authors:** Sachin Tallani, Ritesh Singla, Nishu Singla, Madhumitha Natarajan, Jayaprakash Kukkila

**Affiliations:** 1 Department of Orthodontics & Dentofacial Orthopaedics, Manipal College of Dental Sciences, Manipal, Manipal Academy of Higher Education, Manipal, Karnataka, 576104, India; 2 Department of Public Health Dentistry, Manipal College of Dental Sciences, Manipal, Manipal Academy of Higher Education, Manipal, Karnataka, 576104, India; 3 Department of Dental material, Biomaterials and Research center, Yenepoya Dental College, Mangaluru, Yenepoya Group, Mangaluru, Karnataka, India

**Keywords:** Orthodontic adhesives, Light-cured adhesives, Bracket bonding adhesives, Shear Bond Strength, Adhesive Remnant Index

## Abstract

**Background:**

The dental profession is seeing a constant influx of new adhesive systems from manufacturers, each claiming to be more dependable than the last. This study assessed the bond strength and adhesive remnants of different light-cured adhesives used for bonding metal brackets to teeth.

**Methods:**

80 extracted maxillary premolars with the sound crown structure were acid etched and bonded with brackets on their buccal surfaces utilizing primer and light-cured adhesives into four equal groups, which are Transbond XT, Heliosit, Enlight, and Bracepaste. Shear bond strength (SBS) for de-bonding the brackets were evaluated with Instron- testing machine after 48 hours. The de-bonded samples’ adhesive remnant index (ARI) scores were also measured.

**Results:**

The maximum mean SBS was found for Transbond XT (12.91 ± 2.0 MPa), followed by Bracepaste (12.87 ± 1.59 MPa), Enlight (11.77 ± 1.87 MPa), and lowest for Heliosit (10.93 ± 1.71 MPa). According to the four point scale, adhesive remnant index (ARI), Transbond XT has the least adhesive residue left on the tooth, followed by Heliosit. Enlight and Bracepaste have a similar distribution of adhesive, with both having a maximum amount left.

**Conclusion:**

It can be inferred that all groups involved demonstrated a satisfactory level of bond strength from a clinical perspective. Transbond XT is the preferred orthodontic adhesive over the other three adhesives due to its superior SBS and ARI properties.

## Introduction

Direct bonding procedures, which have become a fundamental technique in modern orthodontics, involve attaching orthodontic appliances to the teeth using adhesives. ^1^ To ensure successful treatment, the bracket-to-tooth bond must be robust enough to sustain the pressures, stresses, and forces exerted during the treatment. The main objective of fixed orthodontic procedures is to achieve optimal bonding between the brackets and the teeth. ^2^ According to Reynolds, force resistances in the 5.9–7.8 MPa range are adequate for withstanding occlusion and orthodontic forces. ^3^ Clinical bracket failure rates vary from 2.7% to 29% for mandibular molars. ^4^ Dislodging brackets during therapy is frustrating for an orthodontist as replacing the dislodged brackets requires additional time and expense and sometimes delays treatment completion. ^5^ Thus, shear bond strength, often known as SBS, is essential when developing bonding materials. ^6^ The type of adhesive used is vital in determining the effectiveness of the bracket’s bond with the enamel surface. Based on Gange’s review, the optimal adhesive for the future should be hydrophilic, removing the requirement for enamel acid etching and exhibiting an SBS value exceeding 20 MPa in dry or wet conditions. ^7^

At the same time, the adhesive material used must not harm the enamel upon removal after the treatment. It’s essential to ensure that all adhesive remains are eliminated to prevent the accumulation of dental plaque along the remnant adhesive interface. ^8^ Failure to do so may lead to an increased risk of tooth decalcification and the development and progression of caries lesions. ^9^ The procedures of de-bonding and cleaning that are necessary after the treatment can consume a considerable amount of time. Furthermore, residual bonding material left on the tooth surface following removal can gradually become discolored, causing patient dissatisfaction. ^10^ An adhesive remnant index (ARI) approach is a qualitative visual scoring method used to assess the adhesive residue left on teeth after de-bonding the brackets. ^11^ Cehreli *et al.* and Kaneshima *et al.* have demonstrated that the ARI visual scoring method is a reliable alternative to scanning electron micrographs (SEM) analysis when evaluating the amount and location of adhesive remnants. ^12^^,^^13^ The ARI method offers a reliable method to evaluate adhesive residue.

When selecting an orthodontic bracket adhesive, it is essential to consider its bond strength and impact on the tooth’s enamel. The dental profession is seeing a constant influx of new adhesive systems from manufacturers, each claiming to be more dependable than the last. ^14^ A perfect orthodontic adhesive should have sufficient bonding strength while preserving the enamel’s natural appearance after removing it from the tooth. ^15^ Researchers have been putting much effort into developing adhesive materials for bonding orthodontic brackets that are both of the highest quality and least invasive. ^16^

The performance of an adhesive agent was evaluated by checking its ability to form a strong bond with a tooth and identifying any adhesive material left on the tooth after removal in a controlled and standardized environment. This *in-vitro* research assessed and compared the SBS (shear bond strength) and ARI (adhesive remnant index) of four light-curing composite adhesives for bonding metal brackets to teeth. The adhesive materials derived from four distinct global manufacturers were Transbond XT (manufactured by 3M Unitek and located in Monrovia, California), Heliosit Orthodontic (manufactured by Ivoclar Vivadent and located in Liechtenstein), Enlight (manufactured by Ormco and located in Glendora, California), and Brace Paste (manufactured by American orthodontics and located in Sheboygan, Wisconsin).

## Methods

**Figure 1.**
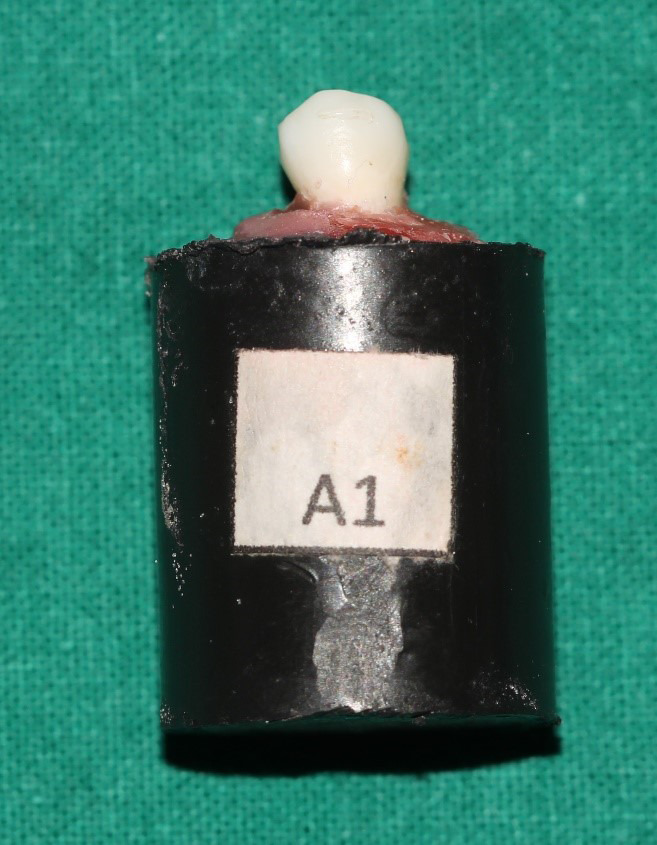
Shows mounted tooth sample.

This was an *in-vitro* study conducted on 80 extracted maxillary premolars. The research’s permission was acquired from the Institutional Ethics Committee of Kasturba Medical College and Kasturba Hospital (IEC 254/2020). 80 intact maxillary premolars with a sound crown structure free of cavities, restorations, or fractures and no developmental anomalies were collected from patients undergoing fixed orthodontic treatment who have advised extraction in their treatment plan. All the teeth that were chosen were scrubbed with pumice that did not include fluoride to remove any debris or stains, and then they were placed in thymol 0.1% solution (weight/volume) until they were used. For experimentation, every tooth was mounted onto a cold acrylic block measuring 19 mm in diameter and 30 mm in height ( Figure 1). The block was created using a cylindrical polyvinyl chloride (PVC) pipe as a template. The tooth roots were buried into the acrylic, and the crown extended outward.

The enamel of each tooth on the buccal surface was first treated with Kerr Gel Etchant 37.5% phosphoric acid for 15 seconds following the manufacturer’s instructions, followed by 10 seconds of rinsing of teeth with water and air drying. The enamel had a noticeable white frosty appearance distributed evenly over the tooth surface. Then, Transbond XT Light Cure Adhesive Primer was used on all the teeth. The primer was put on with a micro brush and cured for ten seconds with a 3M Elipar light curing unit.

Metal brackets were affixed to these teeth with the following adhesive materials marketed by different brands. The teeth were divided randomly into four groups, each with twenty samples.

○ Group A - Transbond XT (3M Unitek, Monrovia, California, USA)
○ Group B - Heliosit Orthodontic (Ivoclar Vivadent, Liechtenstein)
○ Group C - Enlight (Ormco, Glendora, California)
○ Group D - Bracepaste Adhesive (American Orthodontics, Sheboygan, WI, USA)

For this study, 80 mini sprint upper first premolar stainless-steel brackets (McLaughlin Bennett 5.0) marketed by Forestadent were used. As per manufactures guidelines, the dimensions of this bracket base measured 3.0 mm mesio-distal, 3.0 mm occluso-gingival, and 1.9 mm bucco-lingual in height. The calculated bracket base area was 9.0 mm ^2^. According to the MBT prescription, these brackets were fixed onto the labial surface of mounted acrylic teeth. The brackets were coated with adhesive specific to each group and carefully placed in appropriate positions on the teeth. The excess adhesive material was cleaned with a straight probe. The adhesive bonds were cured with a 3M S10 ELIPAR high-power LED light cure device (3M Unitek, Monrovia, CA, USA) with an intensity of 1200 mW/cm ^2^ for 10 seconds, focusing on the gingival and occlusal or incisal features of the brackets. Afterward, the samples were submerged in distilled water at 37 degrees Celsius for 48 hours before de-bonding.

**Figure 2.**
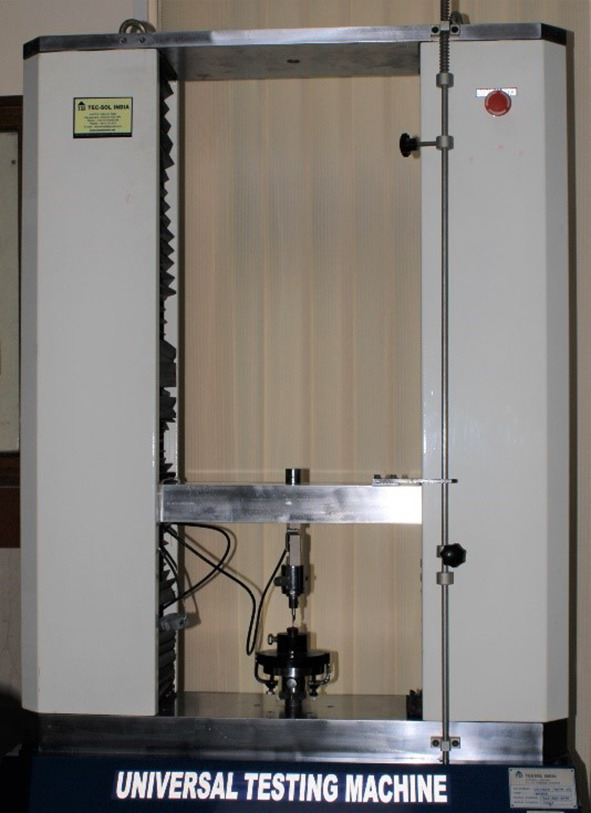
Instron universal testing machine.

**Figure 3.**
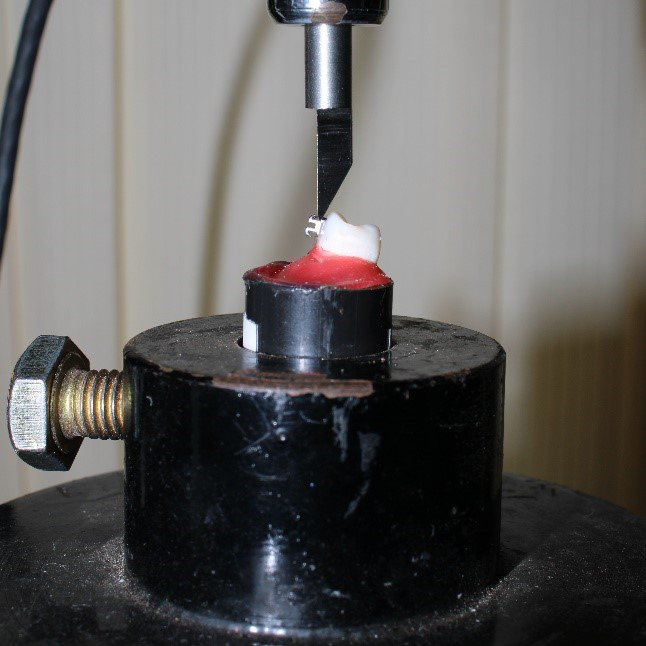
Shear bond strength testing.

In this study, an Instron universal testing machine (Instron, Model no. 3366) ( Figure 2) was utilized to evaluate the shear force needed to detach the brackets fixed on the labial surface of the mounted teeth. Each sample was mounted on the testing machine’s mounting jig to conduct the standardized tests. The acrylic block with mounted teeth with attached brackets was subjected to shear stress ( Figure 3). The base of the brackets was maintained perpendicular to the shear load. The bracket base was subjected to a shear force for de-bonding at a crosshead speed of one millimeter per minute. This was done in an occluso-gingival orientation to simulate the typical forces experienced in the oral cavity. The machine measured the maximum force required to cause the bracket to fracture or de-bond in Newtons (N). To calculate mega-pascals (MPa), divide the value of N by the base area of the bracket (9 mm ^2^).

**Figure 4.**
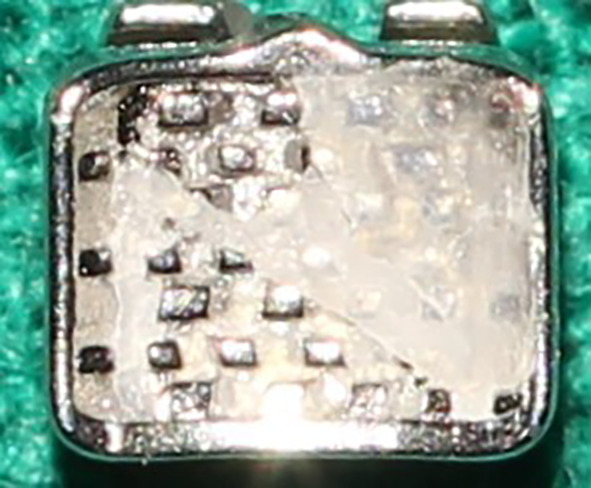
Photograph of bracket base.

**Figure 5.**
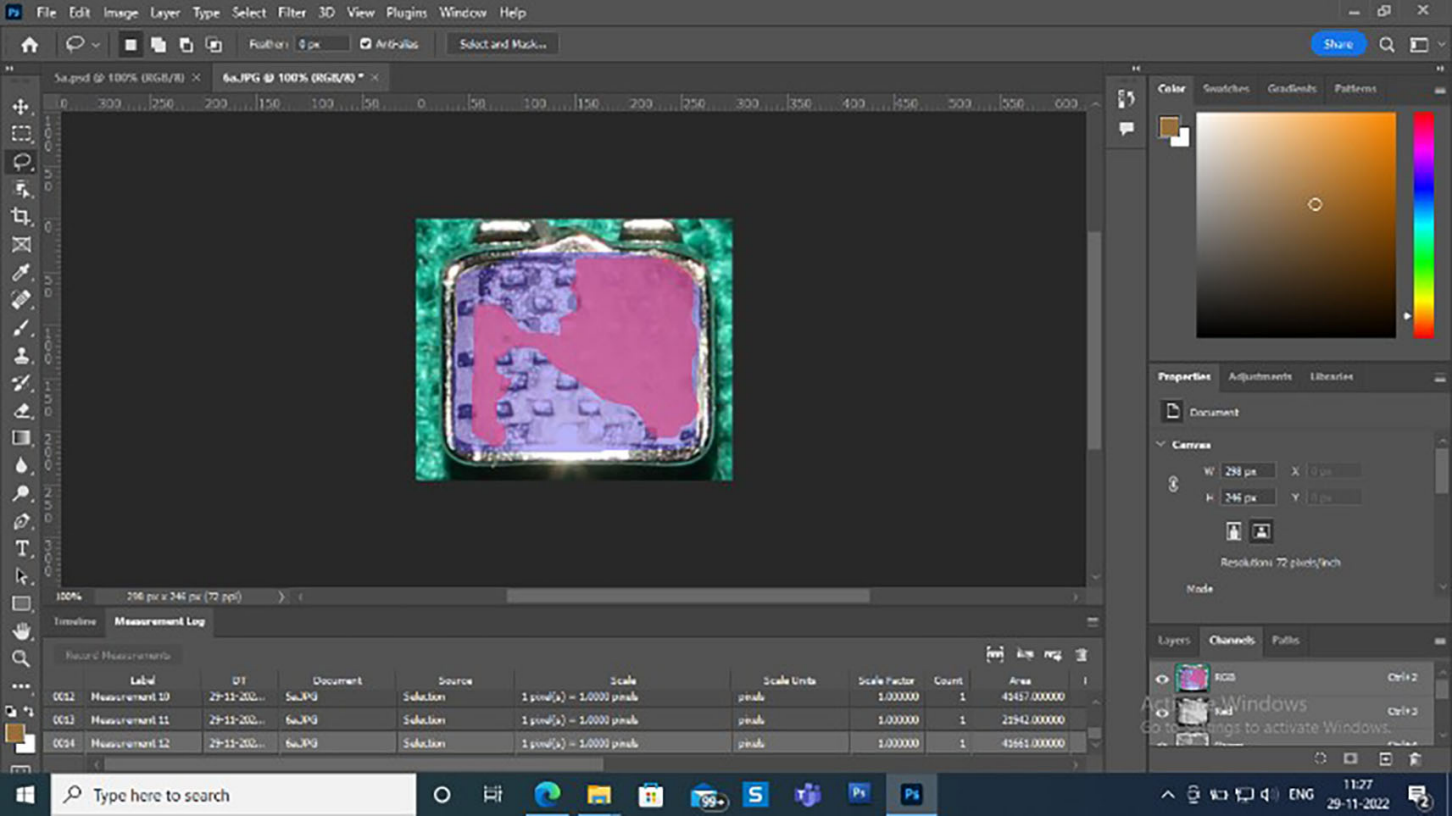
Photoshop software.

Once the tests on the samples had been completed, scaled digital pictures of each bracket base were photographed ^33^ using Canon DSLR 1500 D camera with a 100mm macro lens to take high-resolution photos needed to score ARI ( Figure 4). Standardized images of the bases were taken at a distance of 30 cm and an angle perpendicular bracket base, with particular attention to ensuring that each image depicts the whole bracket base and any residual adhesive. The photos were loaded onto the computer and given arbitrary numbers before being saved as digital photographs in the JPEG file format; following this, the base of the brackets with residual adhesive was scanned. An automated count of the number of pixels was performed to compute the ARI percentages using Photoshop software (Adobe Systems inc, Mountain View, calif). This software program automatically calculated the bracket base’s adhesive residual surface area percentage ( Figure 5). Based on this surface area percentage of residual adhesive, the teeth were assigned a score with Artun, and Bergland ARI on a 4-point scale. The ARI scale states that a score of 0 on the ARI index signifies the absence of adhesive on the enamel. A score of 1 indicates less than 50% of the adhesive remains, and a score of 2 indicates that more than 50% stays on the enamel. Meanwhile, a score of 3 on the ARI index is given when the entire adhesive remains on the enamel.

### Statistical analysis

The study data were explored using the statistical software SPSS 26.0 (SPSS Inc., Chicago, IL) (RRID: SCR_002865). The Shapiro-Wilk test was employed to evaluate the normality of the data. It was found to follow a normal distribution, which enabled parametric tests to be conducted during data analysis. A one-way analysis of variance (ANOVA) and Tukey Posthoc test were conducted to compare the mean SBS Scores (± standard deviation) of the four groups. The comparison of the proportion of discrete ARI scores among the four groups was done with the Chi-square test. A P value less than 0.05 was considered statistically significant. Analyzing the data thoroughly and accurately to draw meaningful conclusions is essential.

## Results

The results of the one-way ANOVA test are presented in Table 1. The study revealed a significant variation in the mean SBS scores across the four groups (p<0.001). The group that used Transbond XT had the highest mean SBS with a value of 12.91 MPa ± 2.0 MPa. The Bracepaste Adhesive group followed closely with a value of 12.87 ± 1.59 MPa. The Enlight group had an SBS of 11.77 MPa ± 1.87MPa, while the Heliosit Orthodontic group had the lowest SBS at 10.93 MPa ± 1.71MPa. Table 2 analyzes the pairwise group comparisons of the mean SBS scores among the groups through the Post hoc test analysis. The mean SBS showed a significant statistical difference between Transbond XT and Heliosit (p = 0.004) and Bracepaste and Heliosit (p = 0.004). The results indicate that Transbond XT and Bracepaste demonstrated significantly higher shear bond strength (SBS) than Heliosit. However, there was no significant difference in the mean SBS values between the Transbond XT, Bracepaste, and Enlight groups.

**Table 1.** Mean shear bond strength (SBS) of each group.

Group	Mean SBS	P value
Transbond XT(A)	12.91 ± 2.0	0.001 *
Heliosit(B)	10.93 ± 1.71	
Enlight(C)	11.77 ± 1.87	
Bracepaste(D)	12.87 ± 1.59	

* A P value less than 0.05 was considered statistically significant.

**Table 2.** Pairwise group comparisons of the mean shear bond strength (SBS) scores.

Posthoc test	P value
A vs B	0.004 *
A vs. C	0.18
A vs. D	0.99
B vs. C	0.44
B vs. D	0.004 *
C vs. D	0.21

* A P value less than 0.05 was considered statistically significant.

The analysis in Table 3 utilized the Chi-Square test to explore the variation in ARI scores across the four groups. According to the study, there was a notable variation in ARI scores between the groups, with a statistical significance of p < 0.0001. In the Transbond XT group, it was discovered that two teeth (10%) had no residual adhesive on the enamel. Half of the teeth in the Transbond XT and Heliosit groups retained less than 50% of the adhesive on the enamel. Among the Enlight and Bracepaste groups, 75% of the samples retained more than 50% of the adhesive on the enamel. However, it was observed that none of the groups had complete retention of the adhesive on the enamel following bracket removal.

**Table 3.** Analysis ofAdhesive Remnant Index (4-Point Scale).

4-Point ARI Scale Score	Transbond XT	Heliosit	Enlight	Bracepaste	P Value
0	2(10%)	0	0	0	0.0001 *
1	9(45%)	10(50%)	5(25%)	5(25%)	
2	9(45%)	10(50%)	15(75%)	15(75%)	
3	0	0	0	0	

* A P value less than 0.05 was considered statistically significant.

## Discussion

An excellent bracket adhesive should have sufficient shear bond strength and be less invasive on the tooth to prevent enamel harm, treatment delays, additional costs, and patient or orthodontist annoyance. This study has analyzed and compared four light cure adhesives from four global manufacturers, *i.e.,* Transbond - XT, Brace Paste, Enlight, and Heliosit. In concurrence with a study, as Reynolds stated, 5.9–7.8 MPa are appropriate to overcome oral forces. ^3^ It has been found that all materials demonstrated optimal bonding strength in the range of 12.91 MPa – 10.93 MPa, capable of withstanding occlusal forces. The study utilized a three-step adhesive procedure, which included conditioning, primer application, and adhesive resin. The Transbond XT primer was included to seal micro-porosity to reduce the risk of demineralization and white spot lesions caused by acid etching on the enamel surface.

The current study quantitatively graded the adhesive residue on bracket bases by image analysis on digital photos. According to the research, the groups showed a notable variation in their ARI scores, which was statistically significant with a p-value of less than 0.0001. It is preferable to have a low Artun ARI score since it specifies that there is minimal residual adhesive remains on the tooth after debonding. ^11^ This would make the finishing and polishing process easier and faster. Additionally, care must be taken to avoid inducing iatrogenic consequences such as cracks, scratches, and the loss of enamel sections while de-bonding the brackets.

Transbond XT is widely used as an orthodontic adhesive agent with a high level of clinical acceptance. ^17^ Extensive research has been conducted on Transbond XT through various studies, proving its superior bond strength and minimal invasiveness on teeth. ^18^^–^^22^ According to this research, the mean SBS for the Transbond XT group was 12.91 MPa ± 2.0 MPa. This finding corresponds with previous studies showing that the SBS of Transbond XT falls within the range of 10.32 MPa to 15.5 MPa. ^20^^,^^21^ Also, in harmony with the literature, the Transbond XT group was found to have the maximum SBS among the four groups. It was the least invasive on the tooth as per adhesive remnant index scores. ^22^ In this group, it was observed that half of the sampled teeth retained less than 50% of the adhesive on the enamel. Additionally, two teeth (10%) had no adhesive retained on their enamel. This group is widely respected as the benchmark and frequently serves as a reference for comparison. ^17^

BracePaste is a relatively new adhesive used in orthodontics. Limited studies have been conducted to analyze its performance and efficiency. ^18^^,^^19^ Based on the current study, both Bracepaste (12.87 ± 1.59 MPa) and Transbond XT (12.91MPa ± 2.0 MPa) demonstrated similar levels of shear bond strength. The findings align with the studies conducted by Katırcıoğlu and Büyükbayraktar, as well as Stephanie Becker, who also observed no notable variance in strength between the two groups. ^23^^,^^24^ However, Shams *et al.* reported that Bracepaste had lower values than Transbond XT. According to a review by Irfan Eser *et al.*, BracePaste and Transbond XT adhesive can be used interchangeably in clinical settings as they demonstrate similar shear stroke numbers. ^25^ According to the manufacturer, BracePaste and Transbond XT possess similar bonding strengths owing to their common Bis-GMA and Quartz Silica ingredients and nearly identical filler content. However, BracePaste adhesive was found to have significantly higher ARI scores in this study, as 75% of the samples retained more than 50% of the adhesive on the enamel. In contrast, Stephanie Becker found that BracePaste was evenly distributed among ARI scores of “1” and “2”. Also, the ARI scores for BracePaste and Transbond XT were significantly similar. ^24^

In the current study, Enlight Group demonstrated an optimal shear bond strength of 11.77 MPa ± 1.87 MPa. There was no statistically significant difference in the mean SBS between the Enlight group and the Transbond XT group, with a value of 12.91 MPa ± 2.0 MPa. Similar results were found in the studies conducted by Shaik M S *et al.*, and Rai S *et al.* ^18^^,^^26^ The ARI scores for Transbond and Enlight adhesives were almost identical in their research. However, in this study, the ARI scores of Enlight group were significantly higher than Transbond XT, with 75% of the samples in this group retaining more than 50% of the adhesive on the tooth. This contrasts with the finding that Verma G *et al.* reported that in most samples, more than 90% of their adhesive or all was left on the brackets. ^27^ During the literature search, only a few studies were discovered that compared the performance of the light cure Enlight group with other existing bonding agents.

According to this study, the Heliosit Orthodontic group exhibited a significantly lower mean SBS (10.93 MPa ± 1.71 MPa) than the other groups included in the research. This aligns with the studies conducted by Shaik M S *et al.* and Rai S *et al.* ^18^^,^^26^ The use of Heliosit as a bonding agent for brackets has not been extensively researched. However, the research findings have consistently demonstrated that Heliosit offers inferior bond strength than Transbond XT. However, it has been established that Heliosit’s bond strengths for orthodontic bonding fall within the recommended range for clinical use. ^28^^–^^31^ The current study found it less invasive on the teeth, similar to Transbond XT. This was evident from the evenly distributed adhesive remnant index scores between ARI scores of “1” and “2”.

## Conclusions

After analyzing the study results, it can be inferred that all groups involved demonstrated a satisfactory level of bond strength from a clinical perspective. The shear bond strength (SBS) was highest for Transbond XT, followed by Bracepaste, Enlight, and lowest for Heliosit. According to the four Point scale (ARI), Transbond XT has the least adhesive residue left on the tooth, followed by Heliosit. Enlight and Bracepaste have a similar distribution of adhesive, with both having a maximum amount left. Therefore, Transbond XT is the preferred orthodontic adhesive compared to the other three adhesives due to its superior SBS and ARI properties. However, it is essential to exercise caution when interpreting the findings of in vitro studies since these tests cannot precisely simulate the conditions within the oral cavity.

## Data Availability

Mendeley Data: Clinical Performance of Light-cured Orthodontic Adhesives for Bonding Brackets – An In-vitro Study, https://doi.org/10.17632/d7ydctfrsf.2. ^32^ This project contains the following underlying data: Clinical Performance of Light-cured Orthodontic Adhesives for Bonding Brackets – Raw data.xlsx (Mendeley Data: Photographs data for Clinical performance of light-cured orthodontic adhesives for bonding brackets – an in-vitro study, https://doi.org/10.17632/fps6fhjdy6.1. ^33^ This project contains the following underlying data:
- Group A (Folder containing all images from group A) - Group B (Folder containing all images from group B) - Group C (Folder containing all images from group C) - Group D (Folder containing all images from group D) Data are available under the terms of the Creative Commons Attribution 4.0 International license (CC-BY 4.0).
