# Supplementary material for: Clinical performance of light-cured orthodontic adhesives for bonding brackets – an in-vitro study
Source: F1000Res. 2023 Nov 7;12:1442. [Version 1] doi: 10.12688/f1000research.141328.1 (PMC10804051; doi:10.12688/f1000research.141328.1)
Supplement: Peer review history

# Reviewer report: Reviewer response for version 1

**DOI:** 10.5256/f1000research.154763.r226513

**Published:** 2024-01-22

**Authors:** Saeed AlSamak

**Affiliations:** 1 Department of Pedodontics Orthodontics and Preventive Dentistry, University of Mosul, Mosul, Iraq

Orthodontic adhesive systems play a pivotal role in the success of bracket bonding during orthodontic treatments. This article evaluates the clinical performance of four commercially available orthodontic adhesives. The previous literature extensively investigated the clinical performances of Transbond XT, Heliosit, Enlight, and Bracepaste regarding shear bond strength and adhesive remanent index. The article well cites the current literature. The study design was appropriate to evaluate the mechanical performances of orthodontic adhesives through shear bond strength and adhesive remnant index. Several commercially available light-cured orthodontic adhesives were selected for this in-vitro study. Standardized brackets were bonded to enamel surfaces, simulating clinical conditions. The specimens were subjected to various assessments, including bond strength measurements, using universal testing machines. The adhesive remnant index was used to evaluate the amount of adhesive remaining on the enamel surface after debonding.

 However, the authors used an alternative way to assess the adhesive remnant index by calculating the amount of adhesive remaining on the bracket instead of the enamel surface, which differs from the original adhesive remnant index described by Artun and Bergland. During the test of shear bond strength, there is a possibility that the orthodontic adhesive will be debonded from both the bracket surface and the enamel surface, which may lead to errors in the calculation of the amount of adhesive remaining on the enamel surface. The statistical analysis was appropriate to reveal the study results. The study revealed significant variations in bond strength among the different adhesive systems tested. The observed differences in bond strength emphasize the need to select orthodontic adhesives carefully based on specific clinical requirements. This in-vitro study contributes valuable insights into the clinical performance of light-cured orthodontic adhesives for bonding brackets regarding shear bond strength and adhesive remnant index.

Is the work clearly and accurately presented and does it cite the current literature?

Yes

If applicable, is the statistical analysis and its interpretation appropriate?

Yes

Are all the source data underlying the results available to ensure full reproducibility?

Yes

Is the study design appropriate and is the work technically sound?

Yes

Are the conclusions drawn adequately supported by the results?

Yes

Are sufficient details of methods and analysis provided to allow replication by others?

Yes

Reviewer Expertise:

Orthodontics, Biomaterials

I confirm that I have read this submission and believe that I have an appropriate level of expertise to confirm that it is of an acceptable scientific standard.

# Reviewer report: Reviewer response for version 1

**DOI:** 10.5256/f1000research.154763.r226515

**Published:** 2024-01-22

**Authors:** Kevser Kurt Demirsoy

**Affiliations:** 1 Department of Orthodontics, Nevsehir Haci Bektas Veli University, Nevsehir, Turkey

This study assessed the shear bond strength and adhesive remnant index of different light-cured adhesives used for bonding metal brackets to teeth. The article is an in vitro study and aimed to evaluate the SBS and ARI scores of different orthodontic adhesive types. Although the subject is far from current, it seems to be designed to evaluate the SBS and ARI scores of the bracket adhesive under the brand name Heliosit, which has not been studied much before. Because there are many SBS and ARI studies conducted in the literature on other equivalent brands. Apart from this, improving the discussion section and adding the limitations section will contribute to the article. Also 33 references are written in the references section, but 31 references are shown in the text. The references section also needs to be revised.

Is the work clearly and accurately presented and does it cite the current literature?

Yes

If applicable, is the statistical analysis and its interpretation appropriate?

Yes

Are all the source data underlying the results available to ensure full reproducibility?

Yes

Is the study design appropriate and is the work technically sound?

Yes

Are the conclusions drawn adequately supported by the results?

Partly

Are sufficient details of methods and analysis provided to allow replication by others?

Yes

Reviewer Expertise:

orthodontics, orthognathic surgery, 3D print systems, Laser in orthodontics

I confirm that I have read this submission and believe that I have an appropriate level of expertise to confirm that it is of an acceptable scientific standard, however I have significant reservations, as outlined above.

# Reviewer report: Reviewer response for version 1

**DOI:** 10.5256/f1000research.154763.r226516

**Published:** 2023-12-29

**Authors:** Vincy Antony

**Affiliations:** 1 MES Dental College, Perinthalmanna, India

1. Overall the article was well-written

 2. In the Methods section, 'cold acrylic block' was mentioned.  Kindly correct/explain the term.

 3. In the abstract methods section, '80 extracted maxillary premolars with the sound crown structure' . Keep it as '80 extracted maxillary premolars with sound crown structure'

 4. The title will have to be changed- Since it is an in-vitro study, the phrase 'clinical performance' cannot be used.

Is the work clearly and accurately presented and does it cite the current literature?

Yes

If applicable, is the statistical analysis and its interpretation appropriate?

Yes

Are all the source data underlying the results available to ensure full reproducibility?

Yes

Is the study design appropriate and is the work technically sound?

Yes

Are the conclusions drawn adequately supported by the results?

Yes

Are sufficient details of methods and analysis provided to allow replication by others?

Yes

Reviewer Expertise:

Orthodontic root response, tooth movement, in vitro studies of bond strength

I confirm that I have read this submission and believe that I have an appropriate level of expertise to confirm that it is of an acceptable scientific standard, however I have significant reservations, as outlined above.
